# Intravenous immunoglobulin treatment with prognosis for the first six months of Guillain–Barré Syndrome in Somalia: Case series

**DOI:** 10.1016/j.amsu.2022.104816

**Published:** 2022-11-05

**Authors:** Nor Osman Sidow, Mohamed Sheikh Hassan

**Affiliations:** Department of Neurology, Mogadishu Somali Turkey Recep Tayyip Erdoğan Research and Training Hospital, Somalia

**Keywords:** Guillain-Barré Syndrome, Intravenous immunoglobulin, Erasmus Gbs Outcome Scale, Modified Erasmus Gbs Outcome Scale

## Abstract

**Introduction:**

Guillain–Barré Syndrome (GBS) is an acute, immune-mediated polyneuropathy that often leads to severe weakness. Intravenous Immunoglobulin (IVIG) is a proven effective treatment for GBS (class 1 evidence). The clinical course of GBS in individual patients is highly variable and difficult to predict.

**Methods:**

It is a retrospective observational study of 10 patients diagnosed with GBS by using nerve conduction studies and lumbar puncture.

**Results:**

Fifty percent of the patients were under 40 years old, and ninety percent were male; all but one were treated with IVIG; and forty percent of the total mEGOS obtained was less than 5. The average predicted probability of being unable to walk unaided after 4 weeks was 47.7%, the average predicted probability of being unable to walk unaided after 3 months was 17%, and the average predicted probability of being unable to walk unaided after 6 months was 8.05%.

**Conclusions:**

Patients presenting with acute ascending weakness should be identified early, and early IVIG treatment for GBS improves disability as measured by The Modified Erasmus GBS Outcome Scale (mEGOS).

## Highlights

• This case series is compliant with the PROCESS Guidelines 2020: http://www.processguideline.com and submit a completed PROCESS 2020 checklist. With attached reference.
• Also we did check the spelling, grammar and syntax and ensured the language is concise.
• Images/figures/photos are suitable anonymised with no patient information or means of identifying the patient.
• Also we included the reviewer comments of statistical software used, rationale for the investigations performed and finding from the study was mentioned at the very start of the discussion.

## 1 Introduction

The Guillain–Barré Syndrome (GBS) is characterized by acute onset of peripheral and cranial nerve dysfunction. Viral respiratory or gastrointestinal infection, immunization, or surgery often precedes neurologic symptoms by 5 days to 4 weeks [1]. Symptoms and signs include rapidly progressive symmetric weakness, loss of tendon reflexes, facial diplegia, oropharyngeal and respiratory paresis, and impaired sensation in the hands and feet [2]. The condition worsens for several days to 3 weeks, followed by a period of stability and then gradual improvement to normal or nearly normal function [3]. Early plasmapheresis or intravenous immunoglobulin (IVIG) (2 g/kg in divided doses) accelerates recovery and diminishes the incidence of long-term neurologic disability [4]. The clinical course of GBS in individual patients is highly variable and difficult to predict. Advanced age is generally reported to be a negative prognostic factor. Previous studies indicate that the difference in severity of GBS can be determined in an early phase of the disease [5]. Peroneal nerve conduction block and age above 40 years turned out to be independent predictors of disability at 6 months [6]. The Modified Erasmus GBS Outcome Scale (mEGOS) at day 7 of admission predict the probability of being unable to walk independently during follow up in patients with GBS [7]. It includes age at onset of neurological symptoms, preceding diarrhea and severity of muscle weakness as defined by the Medical Research Council sum score (MRCsumsScore).

## 2 Materials and methods

The data was a retrospective observational study. To confirm the case of GBS, the Brighton Collaboration (www.brightoncollaboration.org), particularly the Brighton criteria, was used [8]. The records of 9 patients who attended the admission of a tertiary care hospital (Mogadişu Somali Türkiye Recep Tayyip Erdoan Training and Research Hospital) in Mogadishu, Somalia, who were diagnosed with GBS and treated with IVIG were studied. Patients who presented with symptoms of rapidly progressive weakness are the core clinical feature, followed by days to weeks after symptoms of a viral upper respiratory or gastrointestinal infection. Those who were suspected of GBS based on clinical were subjected to nerve conduction studies (prolonged F latency and demyelinating features) and then lumbar puncture. Also, MRI was done to exclude any spinal or brain parenchymal diseases. In patients diagnosed with GBS, IVIG is administered in a regimen of 2 g/kg bodyweight, usually as 0.4 g/kg bodyweight per day for five consecutive days. All the data recorded was transferred and analyzed into a computer database using Statistical Package for the Social Sciences SPSS (version 23.0). This case series has been reported in line with the PROCESS criteria [9]. The World Medical Association's Declaration of Helsinki (2013) states in article 35: ““Every research study involving human subjects must be registered in a publicly accessible database before the recruitment of the first subject."

The study was approved by the medical ethical committee of Mogadişu Somali Türkiye Recep Tayyip Erdoan Training and Research Hospital (2020), and all data were collected from medical records; no harm could potentially be done to the patients, nor did they contain information that could identify individual personal information. The required patient's written informed consent has been waived by the ethical committee.

## 3 Results

Ten patients who had Guillain–Barré Syndrome were included in this study. The mean age was 5 (50%), 9 (90%) were male, 7 (70%) had preceding diarrhea, and 9 (90%) received Intravenous Immunoglobulin (IVIG), except one patient who was not treated with IVIG. (P-value = 0.005) were found to be associated with the prognosis of GBS.

The average predicted probability of being unable to walk unaided after 4 weeks was 47,7%, the average predicted probability of being unable to walk unaided after 3 months was 17%, and the average predicted probability of being unable to walk unaided after 6 months was 8.05%.

Their clinical characteristics were shown in (Table 1).

**Table 1:** Clinical characteristics of 10 patients who had treated with IVIG and their prognosis.

Age (years)	< 40	5 (50%)
	41–60	4 (40%)
	> 60	1 (10%)
Gender	Male	9 (90%)
	Female	1 (10%)
Preceding Diarrhea	Absent	7 (70%)
	Present	3 (30%)
IVIG	Received	9 (90%)
	Not received	1(10%)
mEGOS	Average	5
Predicted probability of being unable to walk unaided after 4 weeks	Average	47.7%
Predicted probability of being unable to walk unaided after 3 months	Average	17%
Predicted probability of being unable to walk unaided after 6 months	Average	8.05%

## 4 DISCUSION

Ten patients were collected from Somali Mogadişu Türkiye Recep Tayyip Erdoan Research and Training Hospital who were diagnosed with GBS in the last two years from 2018 to 2020. Ninety percent of them were treated with IVIG, and their prognosis was measured using the modified Erusmus GBS Outcome Score (mEGOS). Fifty percent of them had a mEGOS of 5. IVIG is a proven, effective treatment for GBS. Not all patients, however, recover enough after a standard IVIG dose. Several clinical factors are associated with the outcome [9]. However, IVIG is an expensive treatment that may induce generally minor side effects. In addition, factors associated with outcome after 6 months are the age of the patient, the presence of preceding diarrhea, and GBS disability score or MRC sum score 1–2 weeks after hospital admission; in addition, a factor to be included now seems to be the magnitude of increase in IgG levels 2 weeks after the start of IVIG [10]^.^

A study published in J ClinImmunol (2010) 30 (Suppl 1): S74–S78 showed a standard dose of IVIG is not sufficiently effective in many GBS patients. Whether these patients might benefit from a second IVIg dose needs further investigation [11].

The average predicted probability of being unable to walk unaided after 4 weeks was 47.7%, the average predicted probability of being unable to walk unaided after 3 months was 17%, and the average predicted probability of being unable to walk unaided after 6 months was 8.05%.

There is a case report about an unusual presentation of Guillain-Barré syndrome (GBS) mimicking stroke in Somlia which is published this year [12].

The limitations of the study are that the cases were too small (10 patients) because there is no tertiary hospital that receives patients presenting with acute progressive ascending weakness and the IVIG is expensive. Another limitation was that there is no plasma exchange (PLEX), which is an alternative treatment for GBS. Data was collected from electronic medical records and it was a retrospective study only.

### 4.1 What is already known on this topic?

• Early intravenous immunoglobulin (IVIG) in GBS accelerates recovery and diminishes the incidence of long-term neurologic disability.
• The clinical course of GBS in individual patients is highly variable and difficult to predict.

### 4.2 What this study adds

• Our study showed the predicted probability of being unable to walk unaided after 4 weeks was 47.7%, after IVIG.
• Patients presenting with acute ascending weakness should be early recognized and should be excluded from other acute flaccid paralysis.

## 5 Conclusion

Early recognition of patients presenting with acute ascending weakness should be early recognized, and early treatment of IVIG for GBS improves the disability by using the modified Erasmus GBS Outcome Scale (mEGOS). Our study showed a 5 score of mEGOS, and the average predicted probability of being unable to walk unaided after 6 months was 8.05%.

## Funding

This article is no funded by any organization or institution, it is granted as case series publication from the hospital.

## Consent for publication

Written informed consent was obtained from the patients for publication of this case series. A copy of the written consent is available for review by the Editor-in-Chief of this journal on request.

## Availability of data and materials

The datasets used and/or analyzed during the current study are available from the corresponding author on reasonable request.

## Guarantor

Nor Osman Sidow, the corresponding author.

## Author contribution

NO involved in patient care and wrote the manuscript, collected data, MSH performed a literature review, and also contributed to the patient care. All authors reviewed and approved the final version for submission.

## Ethical approval

No ethical Approval is needed for this case series.

## Provenance and peer review

Not commissioned, externally peer reviewed.

## Declaration of competing interest

The authors declare no conflict of interest.
